# The Alleviation of Gut Microbiota-Induced Depression and Colitis in Mice by Anti-Inflammatory Probiotics NK151, NK173, and NK175

**DOI:** 10.3390/nu14102080

**Published:** 2022-05-16

**Authors:** Jong-Wook Yoo, Yoon-Jung Shin, Xiaoyang Ma, Young-Hoo Son, Hyo-Min Jang, Chang Kyun Lee, Dong-Hyun Kim

**Affiliations:** 1 Neurobiota Research Center, College of Pharmacy, Kyung Hee University, 26, Kyungheedae-ro, Dongdaemun-gu, Seoul 02447, Korea; hooit96@naver.com (J.-W.Y.); nayo971111@naver.com (Y.-J.S.); xiaoyangma12@gmail.com (X.M.); bitfl@naver.com (Y.-H.S.); jhm0346@naver.com (H.-M.J.); 2 Department of Internal Medicine, School of Medicine, Kyung Hee University, Seoul 02447, Korea; changkyun.lee@khu.ac.kr

**Keywords:** gut dysbiosis, gut microbiome culture, depression, colitis, *Lactobacillus plantarum*, *Bifidobacterium longum*, *Bifidobacterium bifidum*

## Abstract

Gut microbiota dysbiosis is strongly associated with psychiatric disorders and inflammatory bowel disease (IBD). Herein, we examined whether the fecal microbiota of IBD patients with depression (IBDD) and their gut microbiota culture (iGm) could cause depression and colitis in mice and anti-inflammatory probiotics could mitigate depression in iGm-transplanted or immobilization stress (IS)-exposed mice. Fecal microbiota transplantation (FMT) from IBDD patients, which exhibited *Enterobacteriaceae*-rich gut microbiota, and its gut microbiota culture (iGm) increased depression-like behaviors in mice. Their treatments heightened the blood lipopolysaccharide (LPS) level and colonic IL-1β and IL-6 expression. However, FMT from healthy volunteers or sulfasalazine treatment alleviated cGm-induced depressive-like behaviors and hippocampal and colonic inflammation in mice. Moreover, oral administration of *Lactobacillus plantarum* NK151, *Bifidobacterium longum* NK173, and *Bifidobacterium bifidum* NK175, which inhibited LPS-induced IL-6 expression in macrophages, alleviated cGm-induced depression-like behaviors, hippocampal NF-κB^+^Iba1^+^ cell numbers and IL-1β and IL-6 expression, blood LPS, IL-6, and creatinine levels, and colonic NF-κB^+^CD11c^+^ number and IL-1β and IL-6 expression in mice. Treatment with NK151, NK173, or NK175 mitigated immobilization stress (IS)-induced depressive-like behaviors, neuroinflammation, and gut inflammation in mice. NK151, NK173, or NK175 also decreased IS-induced blood LPS, IL-6, and creatinine levels. The transplantation of *Enterobacteriaceae*-rich gut microbiota can cause depression and colitis, as IS exposure, and anti-inflammatory NK151, NK173, and NK175, may alleviate stress-induced fatigue, depression, and colitis by regulating the expression of proinflammatory and anti-inflammatory cytokines through the suppression of gut bacterial LPS.

## 1. Introduction

The gut bidirectionally communicates with the brain through the modulation of the immune, endocrine, and neural systems [1,2]. Inflammatory bowel disease (IBD) is closely connected with the outbreak of psychiatric disorders including anxiety/depression [3,4]. The pathogenesis of IBD is strongly involved in the mucosal immune imbalance toward the gut microbiome as well as host genetic factors [5,6]. Exposure to immobilization stress (IS) or social defeat induces depression with neuroinflammation through the upregulated expression of interleukin (IL)-6 and tumor necrosis factor (TNF)-α in mice, resulting in gut inflammation and dysbiosis [7,8]. Many patients with mood disorders are diagnosed with IBD in clinic studies [9,10]. The disruption of gut microbiota, gut dysbiosis, by exposure to stressors such as pathogen infection and antibiotics, can cause psychiatric disorders with gut inflammation [11,12]. For instance, stress-induced overgrowth of gut *Enterobacteriaceae* and overproduction of gut bacterial lipopolysaccharide (LPS) cause depression with inflammatory bowel disease (IBD)-like colitis in rodents [13,14]. Exposure to *Escherichia coli* or ampicillin causes depression-like behaviors and neuroinflammation in mice through gut inflammation and dysbiosis [7,14,15]. Fecal microbiota transplantation (FMT) from depressive patients causes depression with gut inflammation in mice through the upregulation of IL-6 and TNF-α expression [7,13,16]. Therefore, stressor-induced gut microbiota dysbiosis can be strongly related to the occurrence of psychiatric disorders through the hypothalamus-pituitary-adrenal (HPA) and gut microbiota-gut-brain (MGB) axes.

Probiotics, including Lactobacilli and Bifidobacteria, alleviate gut dysbiosis [17], immune imbalance [18], inflammation [19], depression [20], and memory impairment [21]. *Bifidobacterium adolescentis* IM38 alleviated IS-induced anxiety and high-fat-diet-induced gut inflammation in mice [22,23]. *Lactobacillus reuteri* NK33 and *Bifidobacterium adolescentis* NK98 mitigate stress-induced depression and colitis in mice by the attenuation of gut dysbiosis [16,20]. NVP1704, a combination of NK33 and NK98, alleviates depressive and anxious symptoms in individuals with anxiety, depression, and insomnia [24]. In addition, anti-inflammatory drugs mitigate gut inflammation and depression [25]. Anti-depressant drugs mitigate depression and gut inflammation [26]. These findings suggest that alleviating gut inflammation through the amelioration of gut microbiota dysbiosis may be useful for the treatment of psychiatric disorders.

In the previous studies, the fecal microbiota transplantation (FMT) from patients with IBD and depression (IBDD) is reported to cause depression and gut inflammation in mice [13,27]. Therefore, in the present study, to understand the difference in the occurrence of depression and gut inflammation between IBDD patient fecal microbiota (iFm) and its gut microbiota culture (iGm), we examined their effects on the occurrence of depression and gut inflammation in mice and whether anti-inflammatory probiotics could alleviate gut inflammation and depression in iGm-transplanted mice.

## 2. Materials and Methods

### 2.1. Materials

A general anaerobic medium (GAM) and radioimmunoprecipitation assay (RIPA) lysis buffer were purchased from Nissui Pharm. Co., Ltd. (Tokyo, Japan) and Biosesang Inc. (Seongnam, Korea), respectively. Enzyme-linked immunosorbent assay (ELISA) kits for cytokines were purchased from Ebioscience (Atlanta, GA, USA). Fecal bovine serum (FBS) was purchased from Sigma (St. Louis, MO, USA).

### 2.2. Volunteers

Healthy volunteers (38.2 ± 2.2 years) and patients with IBDD (46.4 ± 15.3 years: ulcerative colitis and depression (UCD), 51.5 ± 17.3 years; Crohn’s disease and depression (CDD), 39.7 ± 11.8 years) were recruited from Kyung Hee University Hospital (KHUP, Seoul, Korea), as in the previous report by Jang et al. [13]. The study protocol and informed consent forms for the stool collection were approved by the Committee for the Care and Use of KHUP Clinical Study (IRB File No., KHUH 2018-03-006-018 and KHUH 2018-12-004-003) and performed according to the ethical principles of the Declaration of Helsinki.

### 2.3. The Preparation of the Fecal Microbiota and Their Gut Microbiota Culture

For the FMT performance, the stools of IBDD patients (UCD, *n* = 4; CDD, *n* = 3) or healthy volunteers (HV, *n* = 3) (0.5 g) were freshly gathered, immediately (<2 h) suspended in sterilized saline (4.5 mL), and the fecal microbiota suspensions were prepared, as previously reported [13]. The colony forming unit (CFU) number of the fecal microbiota suspensions were counted on the GAM agar plates. For the preparation of gut microbiota cultures (iGm: uGm, UCD patient gut microbiota culture; and cGm, CDD patient gut microbiota culture: uGm1 and cGm1, passaged once; and uGm10 and cGm10, passaged ten times), the fresh fecal microbiota suspension (0.1 mL) was inoculated in 50 mL of GAM broth, anaerobically cultured at 37 °C for 20 h, centrifuged (10,000× *g*, 4 °C, 20 min), and washed with saline twice. The resulting precipitate was suspended in saline for the in vivo study, and its CFU number was counted on the GAM agar plates.

### 2.4. Culture of NK151, NK173, and NK175

NK151, NK173, and NK175 were cultured in general media for probiotics such as MRS broth, centrifuged (5000× *g*, 4 °C, and 20 min), washed, and freeze-dried.

### 2.5. Animals

Male C57BL/6 mice (7-weeks old, 19–21 g) were obtained from Koatech Inc. (Seoul, Korea) and kept in plastic cages under the controlled condition (20–22 °C, 40–60% humidity, 12 h light–12 h dark cycle) and fed standard laboratory chow and water ad libitum. Animals were acclimatized for 1 week and used in experiments. All animal experiments were approved by the Institutional Animal Care and Use Committee of Kyung Hee University (IACUC No., KUASP(SE)-20217) and carried out in accordance with the University Guide for Laboratory Animals Care and Usage.

### 2.6. Culture of Peritoneal Macrophages

Peritoneal macrophage cells were isolated, as previously reported [16], and cultured at 37 °C in a 95% air–5% carbon dioxide in RPMI containing 10% FBS and 1% antibiotic-antimycotic solution. For the inflammatory activity assay of gut microbiota, macrophages (1 × 10^6^ CFU/mL) were seeded in a 12-well plate and incubated with gut microbiota suspension (1 × 10^5^ CFU/mL) for 20 h. For the anti-inflammatory activity assay of probiotics, macrophages (1 × 10^6^ CFU/mL) were seeded in a 12-well plate and incubated with probiotics (1 × 10^5^ CFU/mL) in the presence or absence of LPS (100 ng/mL) for 20 h. Levels of cytokines were assayed using ELISA kits (R&D Systems, Minneapolis, MN, USA).

### 2.7. Preparation of Mice with Depression and Colitis (D/C)

D/C mice were made by treatment with the fecal microbiota suspension, its microbiota culture, or IS. Each group consisted of eight or ten mice.

First, to investigate the difference in the occurrence of depression between iFm, which was separated into fecal microbiota of patients with UC and depression (uFm) and fecal microbiota of patients with CD and depression (cFm), and cGm, we orally gavaged uFm, cFm, uGm (the uFm culture), or cGm (the cFm culture, 1 × 10^9^ CFU/mouse/day) in mice daily for 5 days. Depressive-like behaviors were examined.

Second, to examine whether FMT from healthy volunteers or sulfasalazine could alleviate gut microbiota-induced D/C, we prepared mice with cFm-induced depression and examined the effects of healthy volunteer gut microbiota (hFm) or sulfasalazine on depression and colitis. cFm (1 × 10^9^ CFU/mouse/day) was orally given daily for 5 days. hFm (1 × 10^9^ CFU/mouse/day) or sulfasalazine (25 mg/kg/day) was orally given daily for 5 days 24 h after the final treatment with cFm. Depressive-like behaviors were examined.

Third, to evaluate the effects of anti-inflammatory probiotics against gut microbiota-induced depression, we made mice with cFm-induced D/C and examined the effects of probiotics on D/C. cFm (1 × 10^9^ CFU/mouse/day) was treated daily for 5 days, and NK151, NK173, or NK175 (1 × 10^9^ CFU/mouse/day) was orally given daily for 5 days 24 h after the final treatment with cFm. Depressive-like behaviors were examined.

Fourth, to confirm the effects of anti-inflammatory probiotics against depression, we made mice with IS-induced depression by exposing IS daily for 5 days, as previously reported [16], and anti-inflammatory NK151, NK173, or NK175 (1 × 10^9^ CFU/mouse/day) was orally administered daily for 5 days 24 h after the final treatment with IS. Depressive-like behaviors were counted.

### 2.8. Behavioral Tasks

The elevated plus maze task (EPMT) was performed in a plus-maze apparatus, as previously reported [13]. The light/dark transition task (LDTT) was performed in a light/dark box apparatus, as previously reported [16]. The tail suspension test (TST) was performed on the edge of a table, at 30 cm above it, as previously reported [15]. The forced swimming test (FST) was performed in a water-contained transparent plastic jar, as previously reported [16].

### 2.9. ELISA and Limulus Amebocyte Lysate Assays

The tissues of the mouse brain or colon were lysed with RIPA lysis buffer containing a phosphatase inhibitor cocktail and a protease inhibitor cocktail at 4 °C. Levels of cytokines and corticosterone were assayed in their supernatants using ELISA kits [13]. Endotoxin levels were assayed in the blood and stool using a LAL assay kit (Cape Cod Inc., E. Falmouth, MA, USA).

### 2.10. Immunofluorescence Assay

The tissues were immunostained and analyzed by a confocal microscope, as reported previously [15].

### 2.11. Microbiota Sequencing

Genomic DNAs were extracted from human stools and their gut microbiota cultures media using a QIAamp DNA stool mini kit and amplified using the bacterial 16S rRNA V4 region gene-targeted barcoded primers. Their sequences were analyzed using Illumina iSeq 100 (San Diego, CA, USA) [15]. The data availability 16S sequencing dataset (pyrosequencing reads) was deposited in the NCBI’s short read archive under accession number PRJNA827020.

### 2.12. Whole Genome Analysis

The sequencing libraries were carried out according to the manufacturer’s instructions of the 20 kb Template Preparation Using BluePippin Size-Selection System using PacBio DNA Template Prep Kit 1.0. A NK175 genome sequence (1 contig) was described using the PacBio Sequel platform. The whole genome sequence dataset was deposited in the NCBI’s short read archive under accession number PRJNA827022.

### 2.13. Statistics

Experimental data are indicated as the mean ± SEM using GraphPad Prism 9 (GraphPad Software, Inc., San Diego, CA, USA). Significant differences were analyzed using ANOVA and Tukey’s multiple comparisons test (*p* < 0.05).

## 3. Results

### 3.1. iFm and iGm Caused Anxiety/Depression and Colonic Inflammation in Mice

**Figure 1:**
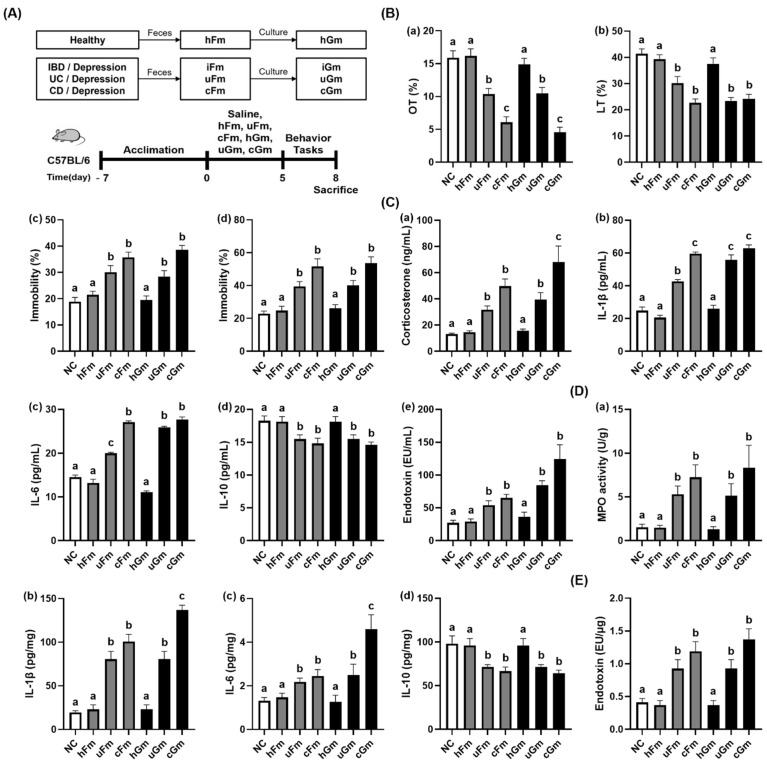
iFm (uFm and cFm) and iGm (uGm and cGm) caused depression and colitis in mice. (**A**) Experimental procedure. (**B**) Effects on depressive behaviors in the EMPT (**a**), LDTT (**b**), TST (**c**), and FST (**d**). (**C**) Effects on blood corticosterone (**a**), IL-1β (**b**), IL-6 (**c**), IL-10 (**d**), and LPS levels (**e**). (**D**) Effects on colonic myeloperoxidase (MPO) activity (**a**), IL-1β (**b**), IL-6 (**c**), and IL-10 levels (**d**). (**E**) Effects on fecal LPS level. Normal control (NC) was treated with saline instead of iFm or iGm. uFm, cFm, uGm, and cGm (1 × 10^9^ CFU/mouse) were orally given daily for 5 days. Groups with same letters are not significantly different (*n* = 10, *p* < 0.05).

First, we examined the effects of iFm (uFm and cFm) and iGm (uGm and cGm) on depression and gut inflammation in mice (Figure 1, Supplement Figure S1). Oral gavage of uFm or cFm significantly reduced time spent in open arms (OT) in the EPMT and time spent in the light compartment (LT) in the LDTT. uFm- and cFm-transplanted mice displayed a decrease in OT to 65.3% and 38.3% (F(6,77) = 25.39, *p* < 0.0001) in the normal control, respectively, and also displayed a reduction in LT to 73.0% and 54.7% (F(6,77) = 18.56, *p* < 0.0001) in the normal control, respectively. Furthermore, treatment with uFm and cFm increased the immobility (depressive behavior) time in the TST to 159.1% and 189.0% (F(6,77) = 17.58, *p* < 0.0001), respectively, and in the FST to 172.8% and 226.7% (F(6,77) = 15.64, *p* < 0.0001) in the normal control, respectively. However, hFm did not significantly increase depressive-like behaviors. Oral gavages of uGm and cGm also increased depressive-like behaviors, while the hGm did not increase it. Oral gavages of uGm and cGm decreased the OT and LT in the transplanted mice. uGm and cGm reduced OT to 66.0% and 28.5% (F(6,77) = 25.39, *p* < 0.0001) and 56.5% and 58.6% (F(6,77) = 25.39, *p* < 0.0001), respectively, compared to the normal control. Treatment with uGm and cGm also increased the immobility time in the TST to 150.3% and 204.4% (F(6,77) = 17.58, *p* = 0.0001) in the normal control, respectively, and in the FST to 175.4% and 235.0% (F(6,77) = 15.64, *p* = 0.0001) in the normal control, respectively. uGm and cGm also increased blood corticosterone, IL-1β, IL-6, and LPS levels, colonic myeloperoxidase activity and IL-1β and IL-6 expression, and fecal LPS level, while colonic IL-10 expression decreased. However, the hFm and its gut microbiota culture hGm did not affect the levels of inflammatory markers in the blood and colon.

**Figure 2:**
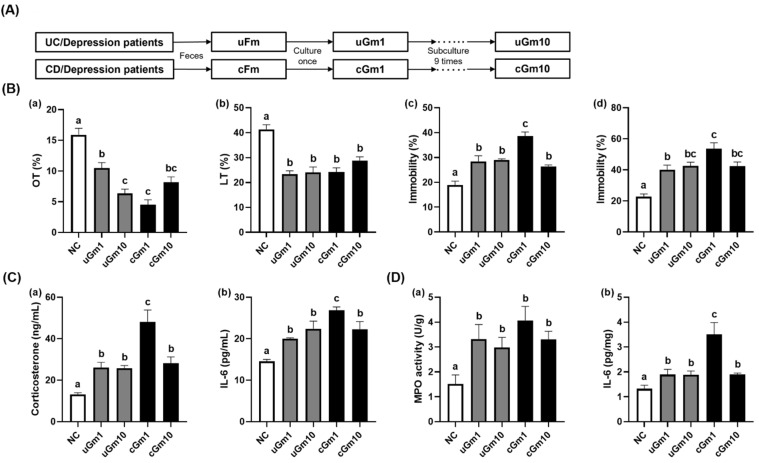
(**A**) Effects of iGm1 and iGm10 on depression and colitis in mice. (**B**) Effects on depressive-like behaviors in the EMPT (**a**), LDTT (**b**), TST (**c**), and FST (**d**). (**C**) Effects on blood corticosterone (**a**) and IL-6 levels (**b**). (**D**) Effects on colonic myeloperoxidase (**a**) and IL-6 expression (**b**). Normal control mice (NC) were treated with saline instead of iGm. iGm (iGm1, 1 × 10^9^ CFU/mouse/day of iGm passaged once; iGm10, 1 × 10^9^ CFU/mouse/day of iGm passaged ten times) were orally given daily for 5 days. Means with same letters are not significantly different (*n* = 8, *p* < 0.05).

Moreover, we investigated the effects of iGm1 (passaged once) and iGm10 (passaged ten times) on the occurrence of depression and colitis in mice (Figure 2, Supplement Figure S2). Treatment with uGm1, uGm10, cGm1, and cGm10 decreased OT to 66.0%, 40.0%, 28.5%, and 51.5% (F(4,48) = 26.17, *p* < 0.0001) in the normal control mice, respectively, and also decreased LT to 56.5%, 58.3%, 58.6%, and 69.6% (F(4,48) = 20.77, *p* < 0.0001) in the normal control mice, respectively. Treatment with uGm1, uGm10, cGm1, and cGm10 increased the immobility time in TST to 150.3%, 153.3%, 204.4%, 139.6% (F(4,48) = 20.52, *p* < 0.0001), respectively, and FST immobility to 175.4%, 186.6%, 235.0%, and 186.0% (F(4,48) = 15.60, *p* < 0.0001), respectively, relative to the NC group. uGm1, uGm10, cGm1, and cGm10 also shortened colon length, increased colonic myeloperoxidase activity, IL-6 expression, blood corticosterone, and IL-6 levels. Overall, the potencies of uGm1 and cGm1 on the occurrence of depression were not significantly different to those of uGm10 and cGm10, respectively.

**Figure 3:**
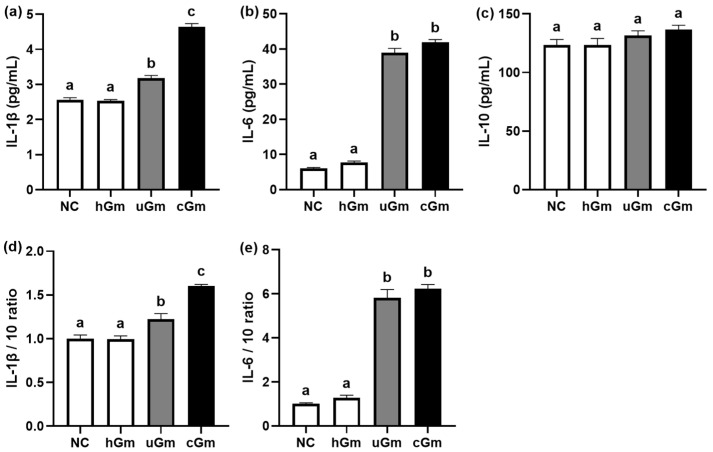
Effect of hGm and iGm on pro-inflammatory and anti-inflammatory cytokine expression in primary macrophages. Effects on IL-1β (**a**), IL-6 (**b**), and IL-10 expression (**c**). Effects on the expression ratios of IL-1β to IL-10 (**d**) and IL-6 to IL10 (**e**). Normal control (NC) was treated with saline instead of hGm or iGm. Test agents (hGm, 1 × 10^5^ CFU/mL of the gut microbiota culture of healthy volunteers; uGm, 1 × 10^5^ CFU/mL of the gut microbiota culture of UC patients with depression; cGm, 1 × 10^5^ CFU/mL of the gut microbiota culture of CD patients with depression) were treated in macrophages for 20 h. Data values are indicated as mean ± SEM (*n* = 4). Groups with same letters are not significantly different (*p* < 0.05).

Next, we investigated the inflammatory activities of hGm and iGm (uGm and cGm) in primary macrophages (Figure 3). Treatment with iGm (uGm and cGm) induced the expression of IL-1β and IL-6 and their ratios to IL-10, while IL-10 expression was not affected. The differences between uGm and cGm were not significant. Moreover, hFm did not significantly affect the expression of IL-1β, IL-6, and IL-10.

**Figure 4:**
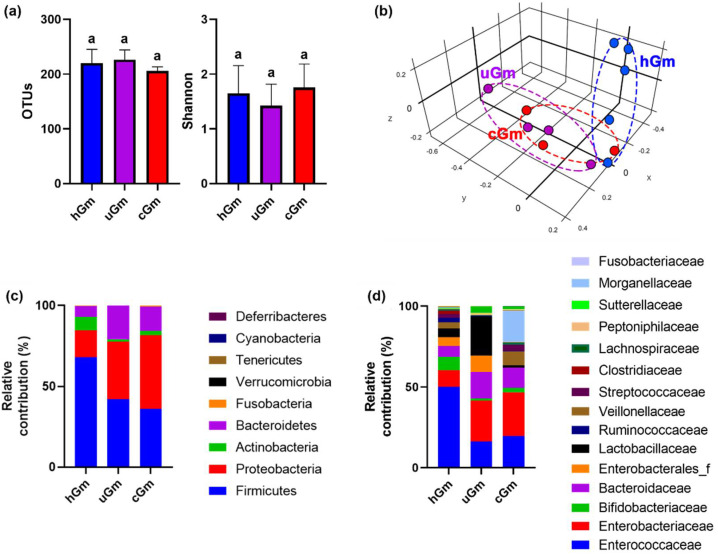
The microbiota composition of hGm and iGm. Effects on α-diversity (OTUs and Shannon index) (**a**) and β-diversity (principal coordinate analysis (PCoA) plot based on BrayCurtis) (**b**). Effect on the microbiota composition at the phylum (**c**) or family levels (**d**). Data values are as mean ± SD (*n* = 8). Groups with same letters are not significantly different (*p* < 0.05).

Next, we analyzed the microbiota compositions of iFm and iGm, using Illumina iSeq 100 (Figure 4). The populations of phylum Proteobacteria (Pseudomonadota) and families *Enterobacteriaceae* and *Muribaculaceae* more highly detected in the iGm (uGm and cGm) than in the hGm, as in the previous report that the populations of Proteobacteria and Enterobacteriaceae were higher in iFm than in hFm [13]. However, their populations between the uGm and cGm were not significantly different.

**Figure 5:**
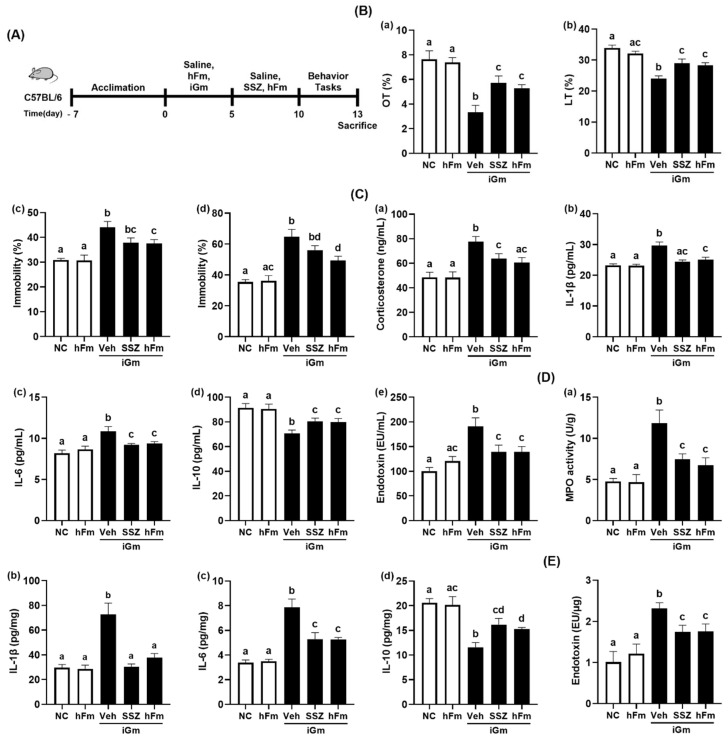
Effects of sulfasalazine and hFm on iGm-induced depression and colitis in mice. (**A**) Experimental procedure. (**B**) Effects on depressive-like behaviors in the EMPT (**a**), LDTT (**b**), TST (**c**), and FST (**d**). (**C**) Effects on corticosterone (**a**), IL-**1**β (**b**), IL-6 (**c**), IL-10 (**d**), and endotoxin (LPS) levels (**e**) in the blood. (**D**) Effects on colonic myeloperoxidase (MPO) activity (**a**), IL-1β (**b**), IL-6 (**c**), and IL-10 levels (**d**). (**E**) Effects on fecal endotoxin level. Normal control mice (NC) were treated with saline instead of test agents and cGm. Test agents (hFm, 1 × 10^9^ CFU/mouse/day of the fecal microbiota suspension of healthy volunteers; SSZ, 25 mg/kg/day of sulfasalazine) were orally given in iGm-treated mice. iGm was treated, as described in Section 2. Groups with same letters are not significantly different (*n* = 8, *p* < 0.05).

Next, we evaluated the effects of hFm and sulfasalazine on cGm-induced depression and colitis in mice (Figure 5, Supplement Figure S3). Oral treatment with sulfasalazine or hFm reduced cGm-induced depression-like behaviors. Oral gavage of iGm increased depressive behaviors: it decreased OT in the EPMT and LT in the LDTT to 43.6% (F(4,25) = 11.12, *p* < 0.0001) and 70.7% (F(4,25) = 14.44, *p* < 0.0001) in the normal control, respectively. Its treatment also increased the immobility time to 142.7 (F(4,25) = 9.03, *p* < 0.0001) in the TST and 183.0% (F(4,25) = 15.18, *p* < 0.0001) in the FST compared to those in the normal control. Treatment with sulfasalazine and hFm alleviated cGm-induced depressive behaviors: sulfasalazine and hFm recovered 75.0% and 69.5% (F(4,25) = 11.12, *p* < 0.0001) in the normal control in the OT, respectively, and 85.4% and 83.5% (F(4,25) = 14.44, *p* < 0.0001) in the normal control, respectively, 122.5% and 121.4% (F(4,25) = 9.03, *p* < 0.0001) in the normal control, respectively, and 158.9% and 139.9% (F(4,25) = 15.18, *p* < 0.0001) in the normal control, respectively. Sulfasalazine and hFm also suppressed cGm-induced IL-6, corticosterone, LPS, IL-1β and IL-6 levels in the blood, while the IL-10 level increased. Furthermore, sulfasalazine and hFm alleviated cGm-induced colitis: they suppressed myeloperoxidase activity, IL-1β and IL-6 expression and increased IL-10 expression. Moreover, sulfasalazine or hFm treatment reduced fecal endotoxin levels.

### 3.2. Probiotics NK151, NK173, and NK175 Suppressed LPS-Induced Expression of Proinflammatory Cytokines in Macrophages

**Figure 6:**
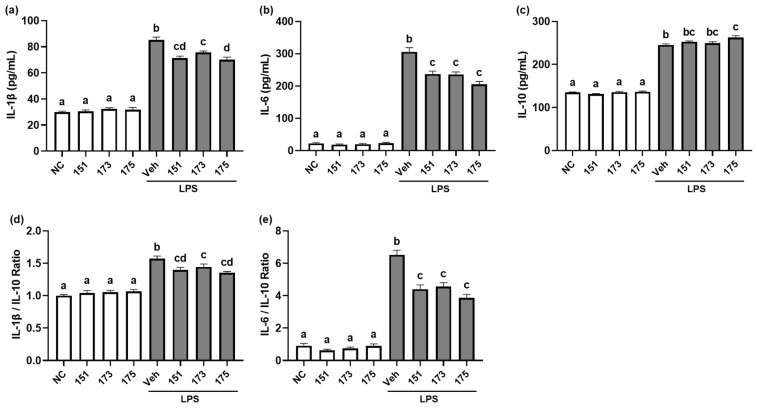
Effect of NK151, NK173, and NK175 on the LPS-induced expression of IL-1β, IL-6, and IL-10 in macrophages. Effect on the IL-1β (**a**), IL-6 (**b**), and IL-10 expression (**c**). Effects on the expression ratio of IL-1β to IL-10 (**d**) and IL-6 to IL-10 (**e**). NC was treated with saline instead of probiotics and LPS. Probiotics (1 × 10^5^ CFU/mL) were treated in macrophages for 20 h. Means with same letters are not significantly different (*p* < 0.05).

We investigated anti-inflammatory effects of probiotics NK151, NK173, and NK175 from the human fecal bacteria collection. Of these, NK151, NK173, and NK175 most strongly suppressed IL-1β and IL-6 expression in LPS-stimulated macrophages, while the expression of IL-10 was weakly induced (Figure 6). NK151, NK173, and NK175 also suppressed the IL-1β to IL-10 and IL-6 to IL-10 expression ratio in LPS-stimulated macrophages.

**Figure 7:**
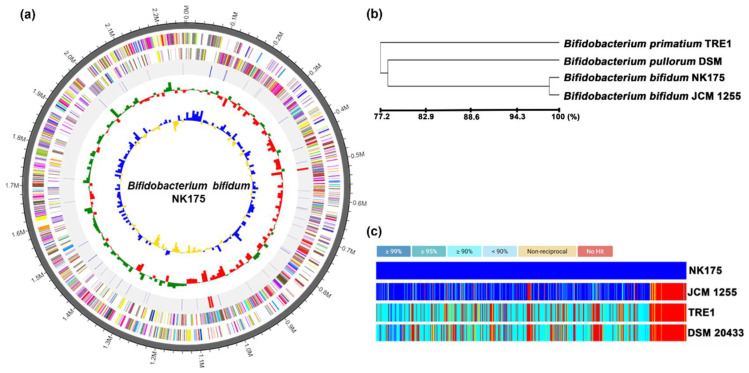
Characterization of NK175 whole genome. (**a**) The pseudochromosome drawn from 1 contig: outermost circle, contig; inner circle, the color coded for the CDS information analyzed in the forward strand; and third circle, the CDS information analyzed in the reverse strand; fourth circle, tRNA (blue) and rRNA (red). (**b**) Neighbor-joining tree based on the OrthoANI distance matrix. (**c**) The pairwise ortholog matrix table.

NK175 was 2,263,214 base pairs with a GC content of 62.9% (Figure 7). The total CDS number was 1052.8. The rRNA and tRNA gene numbers were 9 and 61, respectively. Its genome showed the phylogenetic similarity to *Bifidobacterium bifidum* JCM1255 (98.9%) and *Bifidobacterium pullorum* DSM20433 (79.5%) and was significantly different from those of previously reported NK151 and NK173 [28]. Moreover, they did not show hemolytic activity in the blood agar plate. Therefore, NK175 was identified as *Bifidobacterium bifidum*, based on Gram staining, the API50 CHL kit, and whole genome analyses.

### 3.3. NK151 or NK175 Alleviated cGm-Induced Depression and Colonic Inflammation in Mice

**Figure 8:**
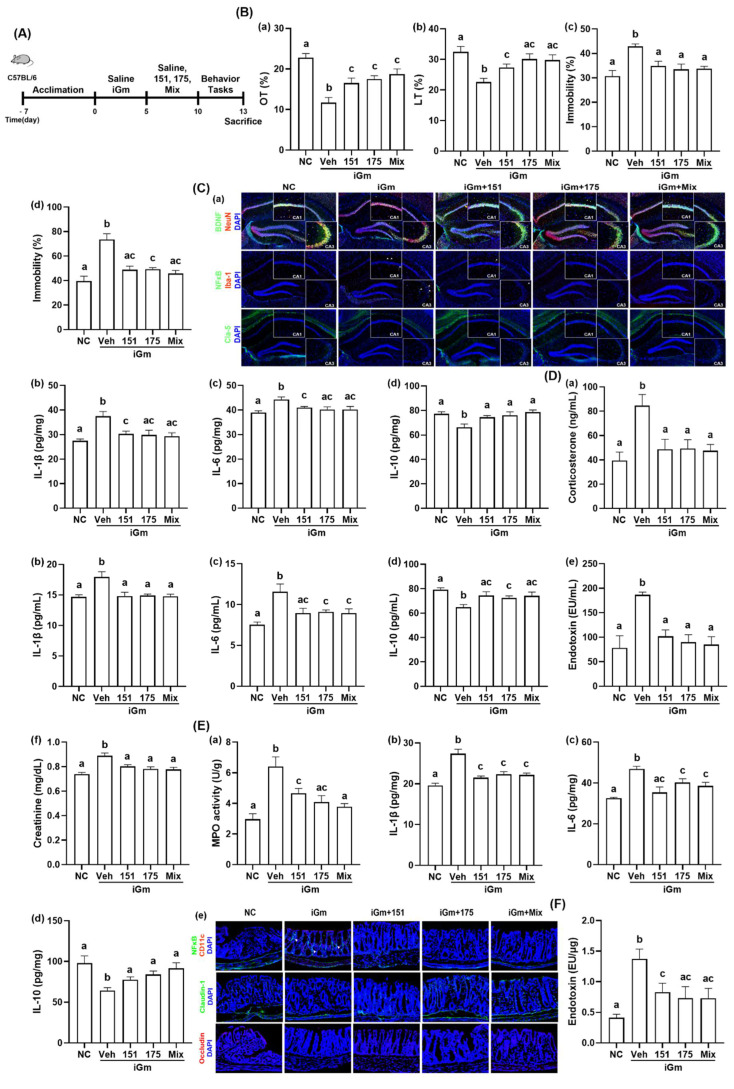
Effects of NK151 and NK175 on iGm-induced depression and colitis in mice. (**A**) Experimental protocol. (**B**) Effects on depressive-like behaviors in the EMPT (**a**), LDTT (**b**), TST (**c**), and FST (**d**). (**C**) Effects on hippocampal BDNF^+^NeuN^+^, NF-κB^+^Iba1^+^, claudin-5^+^ cell populations (**a**), IL-1β (**b**), IL-6 (**c**), and IL-10 values (**d**). (**D**) Effects on blood corticosterone (**a**), IL-1β (**b**), IL-6 (**c**), IL-10 (**d**), endotoxin (**e**), and creatinine levels (**f**). (**E**) Effects on colonic myeloperoxidase (MPO) activity (**a**), IL-1β (**b**), IL-6 (**c**), and IL-10 expression (**d**), and NF-κB^+^CD11c^+^, cladudin-1^+^, and occludin^+^ cell populations (**e**). (**F**) Effects on fecal endotoxin level. Normal control (NC) was treated with saline instead of test agents and iGm. Test agents (veh, vehicle alone; 151, 1 × 10^9^ CFU/mouse of NK151; 173, 1 × 10^9^ CFU/mouse of NK173; 175, 1 × 10^9^ CFU/mouse of NK175) were orally given in iGm-treated mice daily for 5 days. Groups with same letters are not significantly different (*n* = 8, *p* < 0.05).

Next, we investigated whether anti-inflammatory NK151 and NK175 could mitigate iGm-induced depression in mice (Figure 8, Supplement Figure S4). Oral gavage of cGm decreased OT in EPMT to 51.9% (F(4,25) = 11.95, *p* < 0.0001) and LT in the LDTT to 69.7% (F(4,25) = 6.101, *p* < 0.0014) in the normal control and increased the immobility time in the TST to 139.6% (F(4,25) = 7.032, *p* = 0.0006) and in FST to 186.0% (F(4,25) = 15.38, *p* < 0.0001) in the normal control. Oral treatments of NK151, NK175, and their (4:1) mix recovered cGm-suppressed OT to 72.4%, 76.8%, and 82.2% (F(4,25) = 11.95, *p* < 0.0001) in the normal control, respectively, and LT to 84.4%, 93.0%, and 91.8% (F(4,25) = 6.101, *p* < 0.0014) in the normal control, respectively (Figure 2A,B). Their treatments suppressed cGm-induced mobility times in the TST to 113.7%, 109.0%, and 109.9% (F(4,25) = 7.032, *p* = 0.0006), respectively, and in the FST to 123.3%, 124.6%, and 115.4% (F(4,25) = 15.38, *p* < 0.0001) in the normal control, respectively. The combined mix of NK151 with NK175 additively alleviated anxiety/depression-like behaviors. Oral administration of NK151, NK175, or their (4:1) mix increased cGm-suppressed hippocampal IL-10 expression and BDNF^+^NeuN^+^ cell population and decreased cGm-induced hippocampal IL-1β and IL-6 expression and the NF-κB^+^Iba1^+^ cell population (Figure 8C). Treatment with NK151, NK175, or their (4:1) mix also suppressed blood IL-1β, IL-6, endotoxin, and corticosterone expression while increasing blood IL-10 expression. The combined mix of NK151 with NK175 additively alleviated anxiety/depression-like behaviors.

Oral administration of NK151, NK175, or their (4:1) mix suppressed cGm-induced colitis in mice: they suppressed colonic myeloperoxidase activity, IL-1β and IL-6 expression, and the NF-κB^+^CD11c^+^ cell population, while significantly increasing IL-10 expression (Figure 8E). Treatment with NK151, NK175, or their (4:1) mix also suppressed fecal endotoxin levels that were increased with cGm-induced colitis.

### 3.4. NK151, NK173, or NK175 Mitigated IS-Induced Depression and Colonic Inflammation in Mice

**Figure 9:**
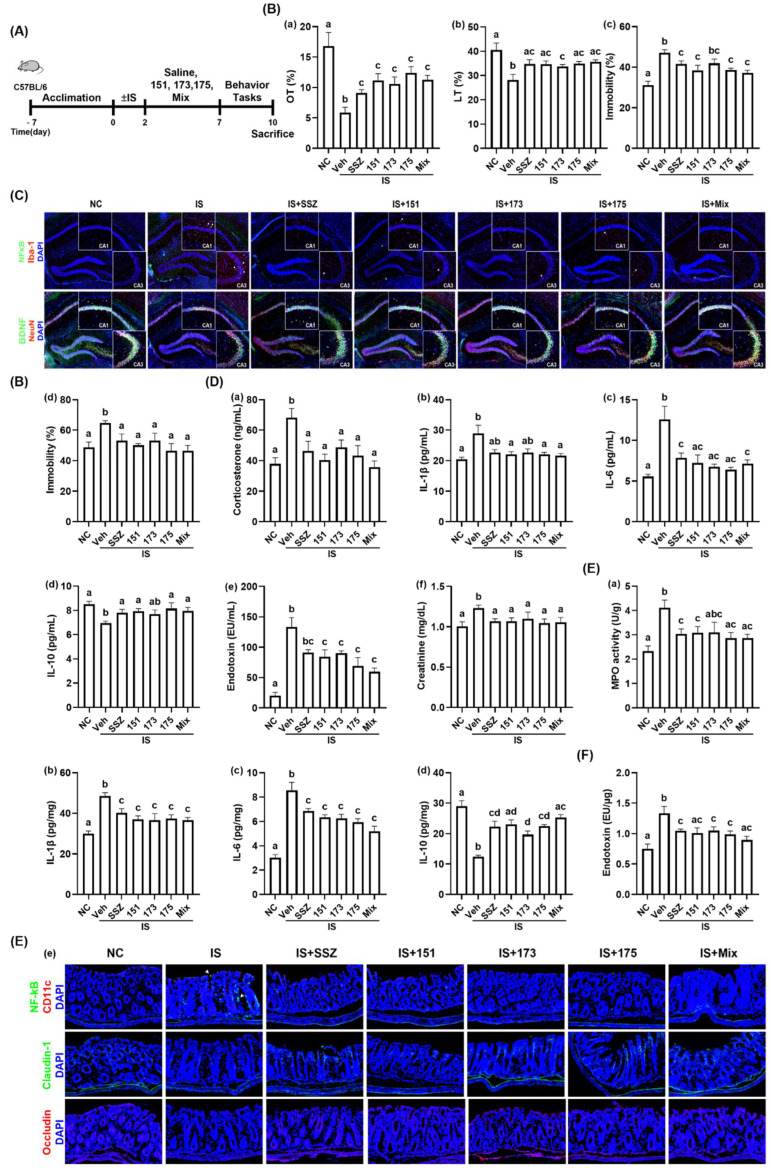
Effects of NK151, NK173, and NK175 on IS-induced depression and colitis in mice. (**A**) Experimental protocol. (**B**) Effects on depressive-like behaviors in the EMPT (**a**), LDTT (**b**), TST (**c**), and FST (**d**). (**C**) Effects on hippocampal BDNF^+^NeuN^+^ and NF-κB^+^Iba1^+^ cell numbers. (**D**) Effects on blood corticosterone (**a**), IL-1β (**b**), IL-6 (**c**), IL-10 (**d**), endotoxin (**e**), and creatinine levels (**f**). (**E**) Effects on colonic myeloperoxidase (MPO) activity (**a**), IL-1β (**b**), IL-6 (**c**), and IL-10 expression (**d**), and NF-κB^+^CD11c^+^, cladudin-1^+^, and occludin^+^ cell numbers (**e**). (**F**) Effects on fecal endotoxin level. Normal control mice (NC) were treated with saline instead of test agents and cGm. Test agents (veh, vehicle alone; 151, 1 × 10^9^ CFU/mouse of NK151; 173, 1 × 10^9^ CFU/mouse/day of NK173; 175, 1 × 10^9^ CFU/mouse of NK175) were orally gavaged. Groups with same letters are not significantly different (*n* = 8, *p* < 0.05).

Next, we examined the effects of NK151, NK173, and NK175 on IS-induced depression in mice (Figure 9, Supplement Figure S5). Exposure to IS significantly reduced the OT in the EPMT to 34.8% (F(6,35) = 7.509, *p* < 0.0001) and LT in the LDTT to 69.3% (F(6,35) = 4.414, *p* < 0.002) to that of the NC mice and increased the immobility times in the TST and FST to 150.1% (F(6,35) = 7.789, *p* < 0.0001) and 132.9% (F(6,35) = 2.961, *p* = 0.0192) in the normal control, respectively. Oral administration of NK151, NK173, NK175, and their (3:1:1) mix increased IS-suppressed OT to 66.3%, 62.6%, 73.6%, and 67.1% (F(6,35) = 7.509, *p* < 0.0001), respectively, and LT 85.3%, 83.4%, 86.1%, and 88.1% (F(6,35) = 4.414, *p* < 0.002) in the normal control, respectively. Their treatments suppressed IS-induced mobility times in the TST 123.2%, 134.2%, 123.6%, and 118.9% (F(6,35) = 7.789, *p* < 0.0001), respectively, and in the FST to 103.1%, 109.3%, 95.5%, and 95.7% (F(6,35) = 2.961, *p* = 0.0192) in the normal control, respectively. The combined mix of NK151, NK173, and NK175 additively alleviated anxiety/depressive-like behaviors. Treatment with NK151, NK173, NK175, or their mix increased IS-suppressed BDNF^+^NeuN^+^ cell population and decreased the IS-induced hippocampal NF-κB^+^Iba1^+^ cell population (Figure 9C). Treatment with NK151, NK173, NK175, or their (3:1:1) mix also decreased corticosterone, endotoxin, creatinine, IL-1β, and IL-6 levels in the blood. On the other hand, NK151, NK173, NK175, and their (3:1:1) mix increased the blood IL-10 level. Furthermore, treatment with NK151, NK173, NK175, or their (3:1:1) mix suppressed IS-induced colitis: they suppressed colonic IL-1β and IL-6 expression and the NF-κB^+^CD11c^+^ cell number along with the fecal endotoxin level, meanwhile increasing the IL-10 cytokine level in the colon.

## 4. Discussion

Gut microbiota bidirectionally communicate with the brain through the MGB and HPA axes [29,30]. FMT from IBD patients raises IBD-like gut inflammation in germ-free mice [27,31]. FMT from IBDD patients raises depression with gut inflammation in specific pathogen-free (SPF) mice [13]. FMT from mice with stress-induced depression also causes depression and gut inflammation with gut dysbiosis in SPF mice [7]. Moreover, most patients with a mood disorder are diagnosed with IBD, and ones with IBD have a mood disorder [32,33]. In addition, patients with IBDD exhibit the higher abundance of gut *Enterobacteriaceae* and *Enterococcaceae* compared to healthy volunteers [13]. For instance, of these gut bacteria, *Escherichia coli*, *Klebsiella oxtytoca*, *Klebsiella pneumoniae*, and *Proteus mirabilis* caused a psychiatric disorder with colitis in vivo [34,35]. These findings imply that gut microbiota dysbiosis may be strongly associated with neuropsychiatric disorders and gut inflammation.

In the present study, treatment with uFm or cFm raised depression with colitis in mice, as previously reported [13]. uFm and cFm also increased the expression of inflammatory markers in the hippocampus, blood, and colon: they induced IL-1β and IL-6 expression and NF-κB activation. Oral gavages of uGm and cGm also induced depressive-like behaviors and inflammation in the hippocampus and colon, similarly with uFm and cFm, respectively. uGm and cGm suppressed the expression of hippocampal BDNF and claudin-5 and colonic claudin-1. However, FMT from healthy volunteers or treatment with sulfasalazine mitigated cGm-induced depression and colitis mice. The potencies of uGm10 and cGm10 serially passaged ten times were not significantly different to those of uGm1 and cGm1 passaged one time, respectively. uGm and cGm exhibited the higher abundance of *Enterobacteriaceae* and *Enterococcaceae* than hGm. These results suggest that uGm and cGm serially passaged up to ten times may be usable to prepare rodents with gut microbiota-induced depression and colitis instead of uFm and cFm, respectively.

We also found that anti-inflammatory probiotics suppressed iGm-induced depressive-like behaviors in mice. Anti-inflammatory NK151, NK173, and NK175 also suppressed iGm-induced inflammation in the hippocampus. However, NK151, NK173, and NK175 upregulated iGm-suppressed hippocampal BDNF and claudin-5 expression and the BDNF^+^NeuN^+^ cell number. NK151, NK173, and NK175 also reduced blood LPS, IL-1β, IL-6, and corticosterone levels. NK151, NK173, and NK175 alleviated iGm-induced colitis: they decreased IL-1β and IL-6 expression, while IL-10 and claudin-1 expression increased. Furthermore, they reduced iGm-induced LPS in the feces. These probiotics also alleviated depressive-like behaviors, neuroinflammation, and colitis in IS-stimulated mice. They decreased IS-induced blood LPS, corticosterone, IL-6, and creatinine levels and the fecal LPS level. In addition, the combined treatment with probiotics additively or synergistically alleviated depressive-like behaviors and inflammation in vivo. Yun et al. reported that oral administration of NK151 and/or NK175 alleviated eye and gut inflammation and gut microbiota dysbiosis in mice with dry eye [36]. We also found that these probiotics increased the iGm- or IS-suppressed expression ratio of IL-1β or IL-6 to IL-10 in the colon and hippocampus. These results imply that anti-inflammatory probiotics including NK151, NK173, and NK175 may alleviate depression, hippocampal and colonic inflammation, and fatigue by the suppression of the IL-1β or IL-6 to IL-10 expression ratio and gut bacterial LPS production.

## 5. Conclusions

The transplantation of *Enterobacteriaceae*-rich gut microbiota can cause depression and colitis, and anti-inflammatory NK151, NK173, and/or NK175 may alleviate stress-induced fatigue, depression, and IBD by modulating the expression ratio of proinflammatory cytokines to anti-inflammatory cytokines and gut microbiota byproducts such as LPS.

## Data Availability

The datasets used and/or analyzed during the current study are available from the corresponding author on reasonable request.

## Supplementary Materials

The following supporting information can be downloaded at: https://www.mdpi.com/article/10.3390/nu14102080/s1, Figure S1. iFm (uFm and cFm) and iGm (uGm and cGm) caused depressive-like behaviors and colitis in mice. Figure S2. Effects of iGm1 and iGm10 on depression and colitis in mice. Figure S3. Effects of sulfasalazine and hFm on iGm-induced depression and colitis in mice. Figure S4. Effects of NK151, NK173, and NK175 on iGm-induced depression and colitis in mice. Figure S5. Effects of NK151, NK173, and NK175 on IS-induced depression and colitis in mice.
