# Supplementary material for: The Alleviation of Gut Microbiota-Induced Depression and Colitis in Mice by Anti-Inflammatory Probiotics NK151, NK173, and NK175
Source: Nutrients. 2022 May 16;14(10):2080. doi: 10.3390/nu14102080 (PMC9147079; doi:10.3390/nu14102080)
Supplement: Supplementary file 1 [file nutrients-14-02080-s001.zip › nutrients-1707543-supplementary.pdf]

[Supplement]

The alleviation of gut microbiota-induced depression and colitis in mice by anti-inflammatory probiotics NK151, NK173, and NK175

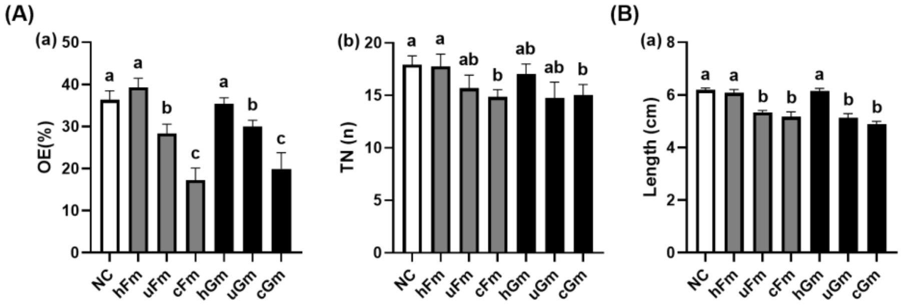

Figure S1. iFm (uFm and cFm) and iGm (uGm and cGm) caused depression-like behaviors and colitis in mice. (A) Effects on depression-like behaviors in the EMPT (a) and LDTT (b). (B) Effects on the colon shortening (a). Data values are as mean ± SEM (n = 10). Means with same letters are not significantly different ($p < 0.05$).

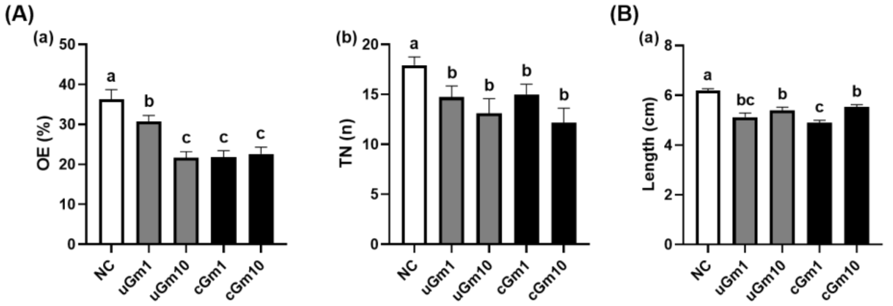

Figure S2. Effects of iGm1 and iGm10 on the occurrence of depression and colitis in mice. (A) Effects on depression-like behaviors in the EMPT (a) and LDTT (b). (B) Effects on the colon length (a). Data values are as mean ± SEM (n = 8). Means with same letters are not significantly different ($p < 0.05$).

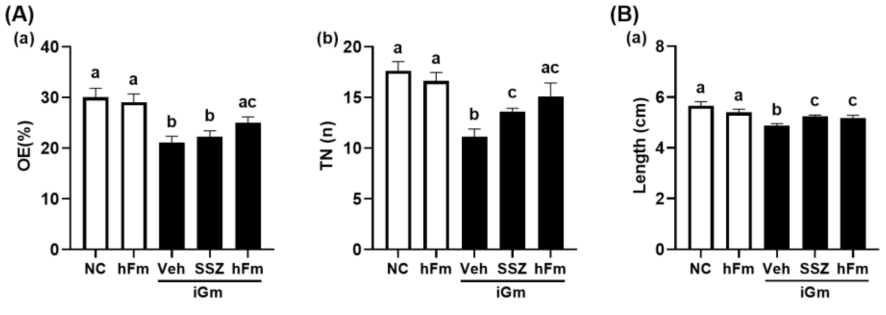

Figure S3. Effects of sulfasalazine and hFm on iGm-induced depression-like behaviors and colitis in mice. (A) Effects on depression-like behaviors in the EMPT (a) and LDTT (b). (B) Effects on the colon shortening (a). Data values are as mean ± SEM (n = 8). Means with same letters are not significantly different ($p < 0.05$).

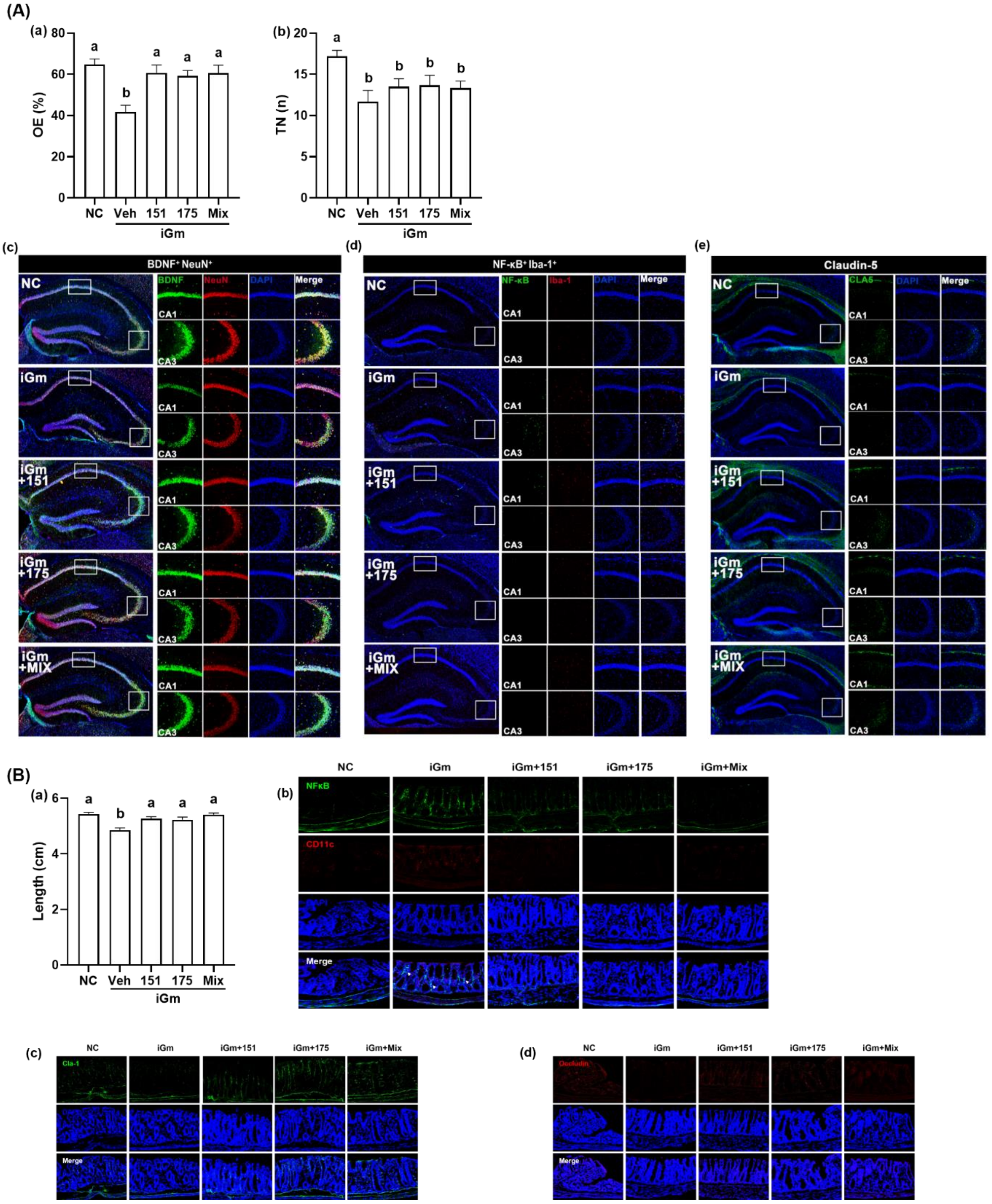

Figure S4. Effects of NK151 and NK175 on iGm-induced depression-like behaviors and colitis in mice. (A) Effects on depression-like behaviors in the EMPT (a) and LDTT (b) and BDNF⁺NeuN⁺ (c) and NF-κB⁺Iba1⁺ (d), and claudin-5⁺ (e) cell populations in the hippocampus. (B) Effects on colon shortening (a), NF-κB⁺CD11c⁺ (b), claudin-1⁺ (c), and occludin⁺ cell populations (d) in the colon. Data values are as

mean ± SEM (n = 8). Means with same letters are not significantly different ($p < 0.05$).

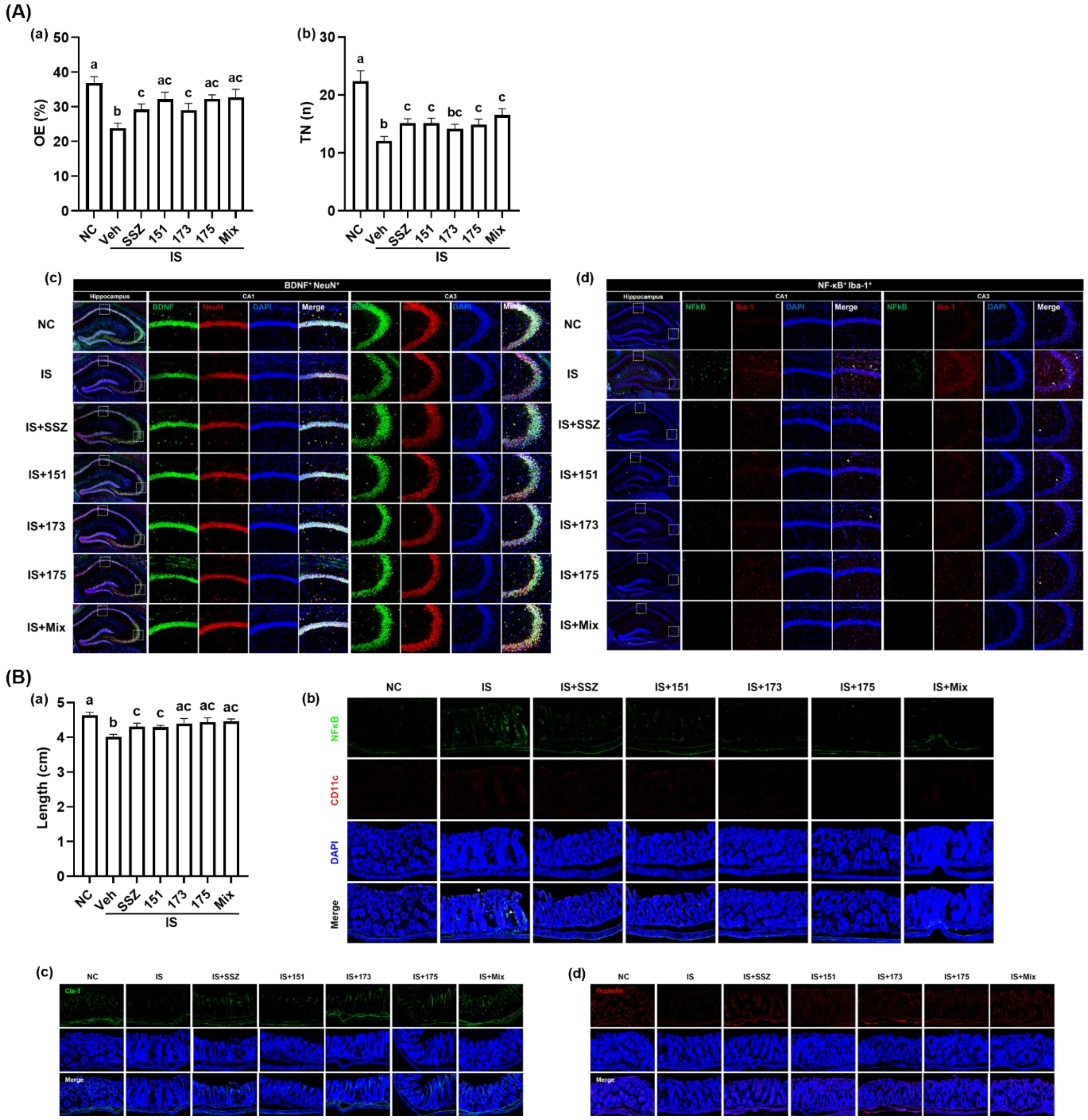

Figure S5. Effects of NK151, NK173, and NK175 on IS-induced depression-like behaviors and colitis in mice. (A) Effects on depression-like behaviors in the EMPT (a) and LDTT (b) and BDNF$^+$NeuN$^+$ (c) and NF-κB$^+$Iba1$^+$ cell populations (d) in the hippocampus. (B) Effects on colon shortening (a), NF-κB$^+$CD11c$^+$ (b), claudin-1$^+$ (c), and occludin$^+$ cell populations (d) in the colon. Data values are as mean ± SEM (n = 8). Means with same letters are not significantly different ($p < 0.05$).
